# Dr James Joseph Cockburn, FRCPsych, FRCPI: Formerly Consultant Psychiatrist, Long Grove and Kingston hospitals

**DOI:** 10.1192/pb.bp.115.052985

**Published:** 2016-08

**Authors:** Ann Cockburn, Olga Bowey-Cockburn

**Figure:**
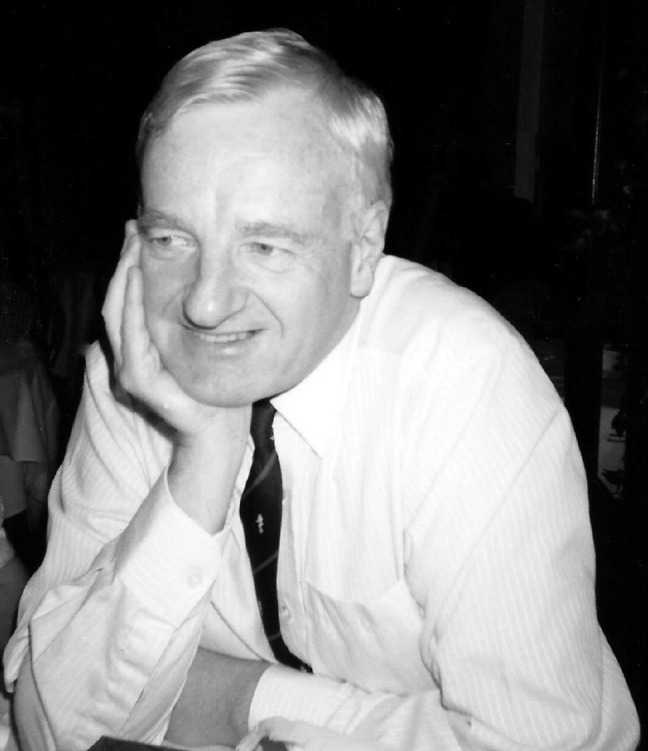


Jim Cockburn, who has died aged 82, was inaugural in several domains of psychiatric practice. He was especially interested in the need to provide patients with treatment and help close to where they lived. Such an idea is taken for granted in the current structure of services, but even as late as the 1960s it was not unusual for patients to be admitted to hospitals many miles away from the family home. Dr Cockburn was instrumental in seeing the catchment area of Long Grove Hospital move from the East End of London to Kingston and Richmond.

His strong scientific leanings also led him to develop a research interest in the condition of spasmodic torticollis. He cogently explored the complexities of the aetiology of the affliction, particularly those involving more psychogenic ideas.

Jim was born in Dublin. His mother died during his first term in medical school at Trinity College, but his father survived long enough to see him graduate in 1955. After a short spell as a locum in general practice, Jim was appointed to the staff of the department of physiology at Trinity College, where he worked happily until 1958. He then started training in psychiatry at St Patrick's Hospital, Dublin. In 1959 he moved to London to continue the development of his psychiatric and psychological skills at the Maudsley Hospital. Here, Jim was determined to maintain his interest in general medicine and attended courses at Guy's Hospital.

He was elected a Fellow of the Royal College of Physicians of Ireland and the Royal College of Psychiatrists; he was equally proud of both. After his appointment to a consultant post at Long Grove and Kingston hospitals in 1964, Jim became very active in management and served for many years as chairman of the psychiatric division. Working in a specialty where teamwork and kindness are important, Jim was respected by his colleagues for both qualities. He remained a consultant at Long Grove and Kingston hospitals until 1999. He was also an active member of the British Medical Association. He continued working as a member of a mental health review tribunal until 2006, several years after retirement from the National Health Service.

As a boy, Jim loved chemistry and was later able to pursue his scientific bent at Trinity and subsequently throughout his career. He applied his scientific training to his hobbies – grafting and growing roses, cultivating strawberries and the production and savouring of beer and wine! He enjoyed travel, theatre, music and watching Ireland play rugby. Most of Jim's family joined him and Olga, his wife, to celebrate his 80th birthday in 2012 in Grassington, Yorkshire, his father's birthplace.

His health gradually failed during the last 2 years of his life. He died peacefully at home with his family around him. He leaves his wife Olga, 4 children and 14 grandchildren. His first wife, Ellen, also survives him.

James Cockburn was born on 28 June 1932 and died on 3 December 2014.

